# Mass Drug Administration for the Elimination of Lymphatic Filariasis — Port-au-Prince, Haiti, 2011–2012

**Published:** 2013-06-14

**Authors:** Thomas Streit, Luccene Desir, Roland Oscar, Jean Frantz Lemoine, Nora Colleen Purcell, Angela Keller, Patrick J. Lammie, Michael Deming, Valery E. Madsen Beau De Rochars

**Affiliations:** Univ of Notre Dame and the Sainte Croix Hospital; Ministry of Public Health and Population; Haiti Centers for Disease Control; Div of Malaria and Parasitic Diseases, Center for Global Health; EIS officer, CDC

Lymphatic filariasis (LF), also known as elephantiasis, results from mosquito-borne infection with filarial worm parasites, predominantly *Wuchereria bancrofti*, and can lead to severe disfigurement from lymphedema and hydrocele. The World Health Organization (WHO) has called for the elimination of LF using the strategy of annual mass drug administration (MDA). WHO defines adequate MDA coverage (the percentage of all residents of an endemic area who swallow the drugs) as ≥65%. By late 2011, all areas in Haiti where LF is endemic had received MDA, except Port-au-Prince, which was considered the most challenging area. The first MDA in Port-au-Prince was conducted from November 2011 through February 2012. To evaluate coverage, a stratified, three-stage cluster-sample survey was conducted. In all, 71% (95% confidence interval = 69%–74%) of persons swallowed the MDA tablets, according to their own or a proxy respondent’s recall. Coverage was highest (77%) among internally displaced persons (IDPs) in camps, and <65% in two of the remaining six survey strata (urban communes). Among the 1,976 adults asked additional questions, 88% said they heard about the MDA before it happened, 74% that they were given tablets, and 71% that they swallowed the tablets. Only 50% of those who did not hear about the MDA in advance swallowed the tablets. The MDA was a large step toward the elimination of LF in Haiti but must be followed by MDA rounds that maintain adequate coverage.

In 2010, WHO estimated that 120 million persons were infected with LF globally (1). In the Americas, Haiti is one of four countries where LF is still endemic, accounting for 78.7% of 12.4 million persons at risk in this region (2). In 2000, WHO called for the elimination of LF by 2020, based on a strategy of annual MDA with drugs that clear microfilaria, the circulating stage of the parasite in humans (3). LF elimination guidelines are based on the expectation that five consecutive annual MDA rounds, each achieving ≥65% coverage in the total population, will result in interruption of transmission (3). By late 2011, at least one round of MDA using albendazole and diethylcarbamazine had been conducted throughout all endemic areas of Haiti except the capital, Port-au-Prince. Port-au-Prince includes the communes of Cité Soleil, Carrefour, Delmas, Pétion-Ville, Port-au-Prince, and Tabarre, and is considered the most challenging area in which to conduct an MDA (4). During November 2011–February 2012, an MDA was conducted for the first time in these communes. Based on reports of doses administered divided by the estimated population of this area, the National Program for the Elimination of Lymphatic Filariasis estimated that 92% coverage had been achieved, varying from 79% to 160% by commune. After the MDA, a household survey was conducted by the Ministry of Public Health and Population and partners as an independent means of assessing coverage and to identify ways of increasing coverage and improving coverage evaluation of MDAs in subsequent years.

A stratified, three-stage cluster sample design was used to select households in seven strata: the IDP camps located within the six communes (one stratum) and non-IDP camp households in each of the six communes (six strata). The first-stage sampling frame for the IDP camps was a list of camps and their sizes in households from administrative records updated every 2–3 months. For non-IDP camp households, the sampling frame was a list of census enumeration areas (sections démographiques d’énumeration [SDEs]), with SDE sizes in households taken from a 2011 update (without enumeration) of the 2003 national census. In all, 35 IDP camps and 30 SDEs in each of the remaining strata were selected, with probability proportional to estimated camp and SDE size. Each selected SDE and camp was divided into two or more segments of approximately equal size in households based on natural lines of division. A single segment was randomly chosen within each selected SDE and camp and survey teams then selected a systematic sample of households within the segment using a sampling interval calculated so that all households in the same stratum had the same overall probability of selection and provided the target sample size.

Within each selected household, a parent or guardian provided responses for children aged <10 years, and this person or another adult provided responses for older children and adults who were absent. Persons asked about swallowing the tablets were first shown the tablets. A knowledge, attitudes, and practices (KAP) questionnaire was administered to persons aged ≥18 years who were present at the time of the survey visit. Coverage and KAP survey data were collected using questionnaires on smart phones and were cleaned and analyzed using statistical software. Children aged <2 years, pregnant women, and severely ill persons were ineligible for treatment during the MDA. However, coverage was defined as the percentage of all persons who swallowed the tablets (3). Coverage estimates for the Port-au-Prince population as a whole (all seven strata) were calculated using sampling weights derived from the overall selection probabilities of households.

A total of 2,102 households were selected for the survey sample during the survey fieldwork, which took place during May 3–21, 2012. In 78% of these households, with a total of 6,345 household members, an adult member was present and agreed to participate in the survey. In all, 63% of persons aged ≥10 years answered the question about swallowing the MDA tablets themselves; for the remaining 37%, the question was answered by a proxy adult household member. In a weighted analysis of all seven strata, the answer to the question about swallowing the MDA drugs was “yes” for 71% (95% confidence interval = 69%–74%), “no” for 23%, and “don’t know” for 6% (Table) of household members in the sample. In all, 97% of “don’t know” answers were from proxy respondents for household members who were absent. “Yes” answers, by stratum, ranged from 60% in Tabarre Commune to 77% in the IDP camps. By this measure, two of the strata, Tabarre and Pétion-Ville Communes, did not achieve adequate (≥65%) coverage. Coverage by sex was nearly the same (71% among females, 72% among males.) Among persons aged ≥2 years, coverage was lowest (55%) among children aged 2–4 years and highest (83%) among children aged 5–14 years, declining gradually in older age groups to 62% overall among persons aged ≥65 years. The coverage-by-age group curve for non-IDP camp residents was slightly lower, but generally paralleled the curve for IDP camp residents, except for the oldest age group, for which non-IDP coverage declined and IDP-camp resident coverage increased (Figure).

A total of 1,976 adults were interviewed with the KAP questionnaire. Because 70% of the respondents were women, who were more often at home than men, the following results were weighted according to selection probabilities and nonresponse rates by gender. In all, 88% of respondents said they heard about the MDA before it began; 74% said they were given tablets during the MDA, and 71% said they swallowed the tablets. Only 50% of those who did not hear about the MDA in advance swallowed the tablets, compared with 74% among those who heard about the MDA in advance. The most commonly mentioned preferred means of communication for those who did not hear about the MDA in advance were television (30%), radio (28%), community resource persons (17%), and a vehicle with loudspeaker (15%).

Most respondents who received tablets got them at a distribution post (85%); less common sites were home (8%) and school (4%). When asked about the distance to the nearest distribution point from their home, 77% of those who did not receive tablets answered that they did not know or were not aware of a distribution point, as compared with 6% of those who received tablets. The most common reason for not swallowing tablets that were received was concern about safety or becoming ill (61%). Among all persons given tablets at a distribution post, 76% swallowed them at the post; 13% reported that no water was available at the post (because of the threat of cholera, the program sought to offer a source of safe drinking water at distribution posts by purchasing water in small plastic bags from commercial sources; persons seeking treatment were given the tablets to swallow at home when distributors ran out of the plastic bags of water). Among all those who swallowed the drugs, 34% reported having adverse events within a day, most often nausea or vomiting (62%), and fatigue (42%).

What is already known on this topic?

Haiti is one of four countries in the Americas where lymphatic filariasis is still endemic. Approximately 9.7 million persons are at risk for lymphatic filariasis in Haiti. By late 2011, at least one round of mass drug administration (MDA) with albendazole and diethylcarbamazine had been conducted in all endemic parts of the country except the capital, Port-au-Prince.

What is added by this report?

A household survey conducted after the first MDA in Port-au-Prince showed that overall coverage with albendazole and diethylcarbamazine was 71% and that five of the seven populations within Port-au-Prince surveyed (residents of six communes and of camps for internally displaced persons) achieved adequate coverage (≥65%). The survey also showed that informing a greater percentage of adults in advance about the MDA and more effectively addressing concerns about safety and side effects might increase coverage. In addition, it showed that coverage estimates for the Port-au-Prince area based on tallies of the number of persons treated and population estimates were inaccurate.

What are the implications for public health practice?

Haiti’s National Program for the Elimination of Lymphatic Filariasis will intensify the dissemination of specific health education messages before subsequent MDAs in Port-au-Prince and rely on household surveys to measure the coverage achieved in the Port-au-Prince area.

## Editorial Note

The 71% MDA coverage calculated by the household survey in Port-au-Prince demonstrates that despite substantial obstacles posed by recent natural disasters and public health emergencies, Haiti has taken an important step toward meeting the challenge of LF elimination. Future MDA efforts should incorporate strategies that were identified in this analysis as potentially important to increase coverage and sustain program success.

MDA coverage, as determined by survey results, was inadequate (<65%) among permanent residents of Tabarre Commune (60%) and Pétion-Ville Commune (62%). This classification is conservative because these communes had the highest proportions of “don’t know” answers to the coverage question (11% and 7%, respectively), the consequence of accepting adults as proxy respondents for household members not available when the survey team visited. If only persons who responded “yes” or “no” are considered, then the coverage estimates for these communes would be ≥65%. For future MDA coverage surveys in Port-au-Prince, survey teams could reduce the percentage of “don’t know” answers by making repeat visits, including in the evening and on subsequent days, if needed, even if doing so within resource constraints requires smaller sample sizes or combining strata.

Although the coverage survey results might have been lowered slightly by “don’t know” answers, they likely present a more accurate estimate of coverage than the 92% derived from reports of doses administered and estimated population sizes. Such estimates of coverage (sometimes called “administrative”) can be in error because of inaccurate denominators, inaccurate reporting of doses administered, and treatment of persons outside their area of residence. The administrative result of 160% for Tabarre Commune clearly reflects one or more of these problems. At present, administrative coverage appears to be too inaccurate to be of value in Port-au-Prince; additional household surveys are planned to track MDA coverage.

Coverage estimates among adult respondents who stated that they heard about the MDA before it began were higher than among those who had not heard about it, suggesting that broadening the reach of pre-MDA communication, including by the means preferred by those who did not hear about the MDA in advance, might increase coverage. The survey also showed that the majority of respondents who did not receive tablets either were not aware of a distribution point or did not know how far away it was. Guidance on narrowing this knowledge gap might be provided by a follow-up study focused on the reasons for the lack of awareness, in particular, on whether post locations were systematically announced by megaphone throughout each post’s catchment area daily during the MDA, as intended. Further efforts to disseminate information on the safety of the drugs also might increase coverage by addressing concerns about safety and becoming ill, which were the most common reasons for not swallowing tablets that had been received. These interventions for increasing coverage might help sustain progress toward national LF elimination. The 2011–2012 MDA in Port-au-Prince demonstrated that Haiti has the capacity to achieve this goal.

## Figures and Tables

**FIGURE:**
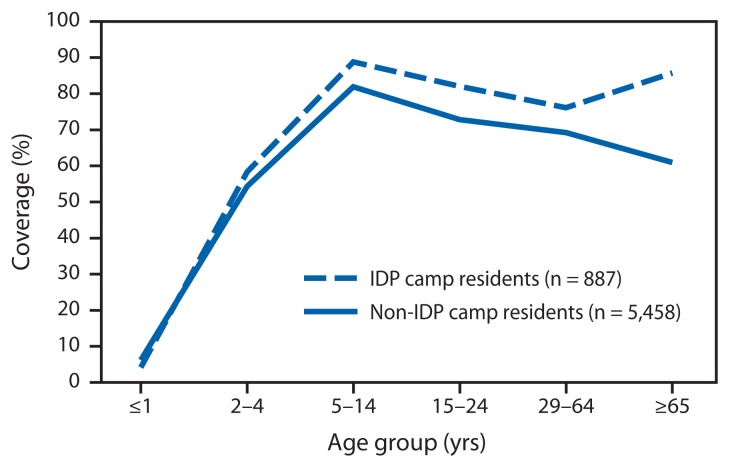
Estimated treatment coverage resulting from mass drug administration for lymphatic filariasis, December 2011–February 2012, by age group and residence in internally displaced person (IDP) camps — household survey, Port-au-Prince, Haiti, May 2012

**TABLE:** Estimated treatment coverage resulting from mass drug administration for lymphatic filariasis during December 2011–February 2012 — household survey, Port-au-Prince, Haiti, May 2012

Survey stratum	“Did you [or name of person for whom respondent answered] swallow tablets for lymphatic filariasis during the last mass drug distribution?” (%)			Sample size

	Yes	No	Do not know	
Carrefour Commune	75	20	5	1,111
Cité Soleil Commune	75	20	4	855
Delmas Commune	71	23	6	829
Pétion-Ville Commune	62	31	7	911
Port-au-Prince Commune	72	22	6	827
Tabarre Commune	60	29	11	925
Internally displaced person camps within the six communes	77	19	4	887
All strata (weighted averages and total)	71	23	6	6,345
